# Liquid Biopsy and Single-Cell Technologies in Maternal–Fetal Medicine: A Scoping Review of Non-Invasive Molecular Approaches

**DOI:** 10.3390/diagnostics15162056

**Published:** 2025-08-16

**Authors:** Irma Eloisa Monroy-Muñoz, Johnatan Torres-Torres, Lourdes Rojas-Zepeda, Jose Rafael Villafan-Bernal, Salvador Espino-y-Sosa, Deyanira Baca, Zaira Alexi Camacho-Martinez, Javier Perez-Duran, Juan Mario Solis-Paredes, Guadalupe Estrada-Gutierrez, Elsa Romelia Moreno-Verduzco, Raigam Martinez-Portilla

**Affiliations:** 1 Department of Reproductive and Perinatal Health Research, Instituto Nacional de Perinatología Isidro Espinosa de los Reyes, Mexico City 11000, Mexico; irmae4901@gmail.com (I.E.M.-M.); salvadorespino@gmail.com (S.E.-y.-S.); djavier40@gmail.com (J.P.-D.); juan.mario.sp@gmail.com (J.M.S.-P.); elsamover@yahoo.com (E.R.M.-V.); 2 Obstetric and Gynecology Department, Hospital General de México Dr. Eduardo Liceaga, Mexico City 06720, Mexico; deyanira_baca@hotmail.com (D.B.); zay.alexi9@gmail.com (Z.A.C.-M.); 3 Iberoamerican Research Network in Obstetrics, Gynecology and Translational Medicine, Mexico City 11560, Mexico; joravibe@gmail.com (J.R.V.-B.); raifet@hotmail.com (R.M.-P.); 4 Maternal-Fetal Department, Instituto Materno Infantil del Estado de Mexico, Toluca 50170, Mexico; dra.rojaszepeda@gmail.com; 5 Laboratory of Immunogenomics and Metabolic Diseases, Instituto Nacional de Medicina Genomica, Mexico City 14610, Mexico; 6 Immunobiochemistry Department, Instituto Nacional de Perinatologia, Mexico City 14080, Mexico; gpestrad@gmail.com

**Keywords:** cfDNA biomarkers, scRNA-seq placenta, extracellular vesicles, placental diagnostics, perinatal technologies

## Abstract

**Background:** Perinatal research faces significant challenges in understanding placental biology and maternal–fetal interactions due to limited access to human tissues and the lack of reliable models. Emerging technologies, such as liquid biopsy and single-cell analysis, offer novel, non-invasive approaches to investigate these processes. This scoping review explores the current applications of these technologies in placental development and the diagnosis of pregnancy complications, identifying research gaps and providing recommendations for future studies. **Methods:** This review adhered to PRISMA-ScR guidelines. Studies were selected based on their focus on liquid biopsy or single-cell analysis in perinatal research, particularly related to placental development and pregnancy complications such as preeclampsia, preterm birth, and fetal growth restriction. A systematic search was conducted in PubMed, Scopus, and Web of Science for studies published in the last ten years. Data extraction and thematic synthesis were performed to identify diagnostic applications, monitoring strategies, and biomarker identification. **Results:** Twelve studies were included, highlighting the transformative potential of liquid biopsy and single-cell analysis in perinatal research. Liquid biopsy technologies, such as cfDNA and cfRNA analysis, provided non-invasive methods for real-time monitoring of placental function and early identification of complications. Extracellular vesicles (EVs) emerged as biomarkers for conditions like preeclampsia. Single-cell RNA sequencing (scRNA-seq) revealed cellular diversity and pathways critical to placental health, offering insights into processes such as vascular remodeling and trophoblast invasion. While promising, challenges such as high costs, technical complexity, and the need for standardization limit their clinical integration. **Conclusion:** Liquid biopsy and single-cell analysis are revolutionizing perinatal research, offering non-invasive tools to understand and manage complications like preeclampsia. Overcoming challenges in accessibility and standardization will be key to unlocking their potential for personalized care, enabling better outcomes for mothers and children worldwide.

## 1. Introduction

The human reproductive system poses significant challenges to perinatal research due to its complexity, particularly in understanding the placenta, maternal–fetal immune interactions, and pregnancy complications [1]. The placenta, as a central organ in maternal–fetal health, plays a critical role in nutrient exchange, gas transfer, immune modulation, and endocrine regulation. However, its inaccessibility during pregnancy and the limitations of existing experimental models have historically hindered progress in understanding the mechanisms underlying pregnancy-related disorders [2].

Traditional research approaches are constrained by the invasive nature of tissue sampling and the inability of animal models to fully replicate human placental physiology. This has limited the development of accurate diagnostic tools and therapeutic strategies for conditions such as preeclampsia, preterm labor, and fetal growth restriction, which significantly contribute to maternal and perinatal morbidity and mortality. Emerging technologies, such as liquid biopsy and single-cell analysis, have introduced non-invasive and high-resolution methods to investigate these challenges [3]. Liquid biopsy enables the analysis of cell-free DNA (cfDNA), RNA (cfRNA), and extracellular vesicles (EVs) in maternal blood, providing molecular insights into placental function and maternal–fetal communication. Single-cell RNA sequencing (scRNA-seq) offers detailed characterization of placental cell populations, revealing their specific roles in physiological and pathological processes [4]. These technologies have demonstrated potential to enhance the detection of pregnancy complications and deepen understanding of placental biology. However, their clinical applications remain limited due to barriers such as cost, technical complexity, and the lack of standardized protocols. Furthermore, research into their diagnostic accuracy, reproducibility, and relevance across diverse populations is still evolving.

This review systematically examines the current applications of liquid biopsy and single-cell analysis in perinatal research, with a focus on placental development and pregnancy complications. By identifying key findings, knowledge gaps, and methodological challenges, this review aims to provide a framework for advancing research and clinical practice in this field.

**RESEARCH QUESTIONS**

What are the current applications of liquid biopsy and single-cell analysis in studying placental development and perinatal complications?

What evidence supports the diagnostic utility of these technologies for early identification of complications such as preeclampsia, preterm birth, and fetal growth restriction?

What knowledge gaps remain in the research, and how might future studies address them?

## 2. Materials and Methods

### 2.1. Eligibility Criteria

Studies were selected for inclusion based on relevance to the use of liquid biopsy or single-cell analysis within perinatal research, with a particular focus on studies addressing placental development and pregnancy complications. Eligible studies specifically explored diagnostic, monitoring, or therapeutic applications related to conditions such as preeclampsia, preterm birth, and fetal growth restriction. Only original research articles, reviews, and systematic reviews published in peer-reviewed journals were included to ensure methodological rigor. Studies centered solely on animal models, conference abstracts without full-text availability, or publications in languages other than English were excluded to maintain the review’s focus on clinically applicable human research.

### 2.2. Search Strategy

The review employed a comprehensive search strategy across major databases, including PubMed, Scopus, and Web of Science. The search terms combined controlled vocabulary, such as Medical Subject Headings (MeSH), and free-text keywords to capture a broad spectrum of relevant studies. Keywords included terms such as “liquid biopsy”, “cell-free DNA”, “cfRNA”, “single-cell RNA sequencing”, “placental development”, “perinatal complications”, “preeclampsia”, “preterm birth”, and “fetal growth restriction”. Boolean operators were applied to refine the search and filters were used to limit the results to studies published within the last ten years to focus on recent advancements (Table S1). The search strategy included specific terms related to extracellular vesicles and exosomes, which allowed the identification of relevant studies assessing their role in placental disorders such as preeclampsia.

This scoping review adhered to the PRISMA-ScR (Preferred Reporting Items for Systematic Reviews and Meta-Analyses extension for Scoping Reviews) guidelines, ensuring a systematic and transparent approach to identifying, evaluating, and synthesizing the literature (Table S2, Figure S1). In addition to the 13 studies included through the systematic search and screening process, 19 additional peer-reviewed publications were manually identified through expert consultation and citation tracking. These references were not retrieved from the original search strategy but were included to enrich the narrative synthesis with recent advancements, particularly in areas underrepresented in the initial results. This hybrid approach aligns with the flexible nature of scoping reviews and ensures a more comprehensive and up-to-date overview of the field.

To increase openness and transparency, this scoping review has been registered on the Open Science Framework (OSF) https://doi.org/10.17605/OSF.IO/DVHT3.

### 2.3. Study Selection

Once retrieved, the studies were imported into a reference management system to eliminate duplicates. Two independent reviewers (JTT and DB) then screened titles and abstracts according to the eligibility criteria, followed by a full-text assessment for studies that met the initial criteria. Discrepancies between reviewers were discussed and resolved, with a third reviewer consulted if needed to ensure consistency in study selection.

### 2.4. Data Extraction

Data from the selected studies were systematically extracted using a standardized form developed for this review. The extracted information included study characteristics (such as author, year, journal, and study design) to capture basic metadata, and details about the type of technology applied (e.g., liquid biopsy, single-cell RNA sequencing, or organoid models). Data were also collected on each study’s target outcome, including applications in diagnostic biomarker identification, gene expression profiling, or therapeutic implications. Additionally, the main findings related to perinatal health were summarized, focusing on how the technology was applied in placental development or used to identify biomarkers for complications.

### 2.5. Data Synthesis and Analysis

The extracted data were synthesized descriptively, with studies organized into thematic categories to address the review’s research questions. Key themes included diagnostic applications, methods for monitoring placental health, and biomarker identification relevant to pregnancy complications. The analysis provided an overview of current research applications, while also highlighting limitations and research gaps within the field (Table S3).

### 2.6. Limitations

The scope of this review was limited by the exclusion of non-English studies, which may reduce the global applicability of the findings. Additionally, as liquid biopsy and single-cell analysis technologies continue to evolve, new studies may quickly emerge, potentially extending or refining the insights presented here.

## 3. Results

### 3.1. Overview of Included Studies

This scoping review identified 12 studies that investigated the applications of advanced technologies in perinatal research. These studies explored the utility of liquid biopsy, scRNA-seq, organoid models, and EVs in understanding placental biology and pregnancy complications. The included research encompassed both basic and applied science, ranging from detailed molecular studies of placental cell interactions to translational applications in diagnosing conditions such as preeclampsia, preterm labor, and fetal growth restriction. Collectively, the studies demonstrated the transformative potential of these technologies in enhancing our understanding of maternal–fetal interactions, improving diagnostic capabilities, and exploring new therapeutic strategies for pregnancy-related complications. These findings highlight not only the promise of emerging tools but also the gaps and limitations that require further investigation to fully realize their clinical potential.

### 3.2. Role of Emerging Technologies in Understanding Placental Biology and Pregnancy Complications

Emerging technologies such as liquid biopsy and single-cell analysis have significantly advanced our understanding of the placenta’s biology and its role in pregnancy complications. The placenta, as a central organ in maternal–fetal health, governs the exchange of nutrients, gases, and waste, while orchestrating a dynamic network of immunological and hormonal signals. However, traditional approaches to studying placental function are limited by invasive sampling methods and the lack of reliable experimental models. These emerging technologies have significantly advanced our understanding of placental biology and maternal–fetal interactions, providing valuable tools for diagnosing and monitoring pregnancy complications. Table 1 summarizes the key technologies, their applications, notable findings, and current limitations. This overview highlights both their potential and the challenges that must be addressed to fully integrate these tools into perinatal research and clinical practice.

Liquid biopsy provides a non-invasive avenue to access molecular information from the placenta through maternal blood. Analyzing cfDNA and cfRNA offers a real-time view of placental gene expression, enabling researchers to detect subtle changes associated with dysfunction. For example, fragmentation patterns in cfDNA reflect disruptions in placental cellular activity, offering insights into the early stages of complications such as placental insufficiency. Similarly, EVs, which carry microRNAs and proteins of placental origin, illuminate key signaling pathways in maternal–fetal communication. These molecular messengers facilitate the investigation of processes like trophoblast invasion and immune modulation, which are crucial for a successful pregnancy and often impaired in conditions like preeclampsia.

scRNA-seq further enhances our ability to dissect the cellular and molecular complexity of the placenta. By resolving individual cell populations, this technology identifies the cellular diversity and specialized roles within the placenta. For instance, scRNA-seq studies of placental pericytes have uncovered pathways critical for vascular remodeling, a process essential for fetal growth. Additionally, the use of trophoblast organoids, which simulate early placental development, has provided a controlled platform for investigating the mechanisms of hormone production and nutrient transport. These advancements collectively enable a mechanistic understanding of placental biology, linking molecular findings to clinical outcomes and paving the way for targeted interventions.

#### Technical Distinctions Between Liquid Biopsy and Single-Cell Technologies

While both liquid biopsy and single-cell analysis have demonstrated remarkable utility in perinatal research, they differ substantially in methodology, resolution, and clinical applicability.

Liquid biopsy is a non-invasive technique based on the analysis of circulating biomolecules—including cfDNA, cfRNA, and EVs—present in maternal blood. These molecules originate from placental or fetal tissues and are released through apoptosis, necrosis, or active secretion [5,6,7]. Their analysis provides a dynamic, systemic overview of placental gene expression and function, making liquid biopsy particularly suitable for real-time monitoring and early detection of complications such as preeclampsia or placental insufficiency [8,9].

In contrast, single-cell technologies such as scRNA-seq offer a high-resolution, localized view of placental biology [10,11]. These methods require fresh tissue samples, which are enzymatically dissociated into single-cell suspensions before sequencing [12,13]. This approach allows for the identification of distinct cell types, differentiation trajectories, and cellular interactions within the placenta, offering mechanistic insights into processes like trophoblast invasion and vascular remodeling [14]. However, scRNA-seq demands significant bioinformatic expertise and infrastructure, limiting its current clinical applicability [15].

### 3.3. Applications of Liquid Biopsy for Perinatal Complications

**Figure 1:**
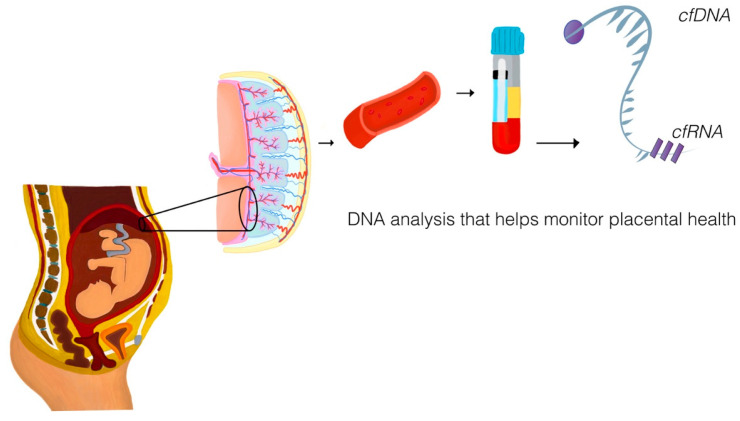
Liquid biopsy for placental health analysis in perinatology.

Liquid biopsy technologies have emerged as a revolutionary, non-invasive diagnostic approach for assessing maternal and fetal health (Figure 1). By analyzing maternal blood, these technologies enable the detection of cell-free nucleic acids, including cfRNA and cfDNA, and other molecular components, such as EVs, that provide critical insights into placental function and pregnancy complications. To address specific associations between molecular findings and pregnancy complications, this section summarizes the experimental evidence linking liquid biopsy biomarkers to conditions such as preeclampsia, preterm birth, fetal growth restriction, and placenta accreta spectrum.

A large prospective study identified a panel of 18 cfRNA genes detectable at between 5 and 16 weeks’ gestation that predicted later development of preeclampsia with ~75 % sensitivity well before clinical onset. These cfRNA changes were enriched for transcripts related to endothelial and immune pathways implicated in disease pathophysiology [6,16].

One study examined the utility of cfRNA biomarkers in detecting preterm labor. It found that certain inflammation-associated transcripts were significantly elevated in maternal blood during late gestation in cases of preterm labor. These findings suggest that cfRNA could serve as an early warning system, allowing clinicians to identify pregnancies at risk for preterm birth and implement timely interventions to prevent adverse outcomes [17]. Another study focused on cfDNA and its ability to reflect placental gene expression patterns. Fragmentomics of cfDNA in maternal plasma reflect placental gene regulation networks. Altered fragmentation patterns correlated with genes involved in angiogenesis and trophoblast function, suggesting utility for early detection of placental insufficiency and risk of FGR [18,19].

This figure illustrates the pathway of placental molecule release and their subsequent analysis through liquid biopsy, an innovative and non-invasive tool in perinatal research. During pregnancy, the placenta releases cell-free DNA (cfDNA) and RNA (cfRNA) into the maternal bloodstream via cellular processes such as apoptosis, necrosis, and active secretion. These circulating molecules are obtained through maternal peripheral blood sampling and analyzed using advanced laboratory techniques, including nucleic acid sequencing. Liquid biopsy facilitates dynamic monitoring of placental function, offering molecular insights into critical processes such as vascular remodeling, trophoblast invasion, and immune regulation. As discussed in this scoping review, liquid biopsy represents an emerging technology with the potential to address knowledge gaps in placental pathophysiology and improve the early detection of pregnancy complications, such as preeclampsia, fetal growth restriction, and preterm birth.

**Figure 2:**
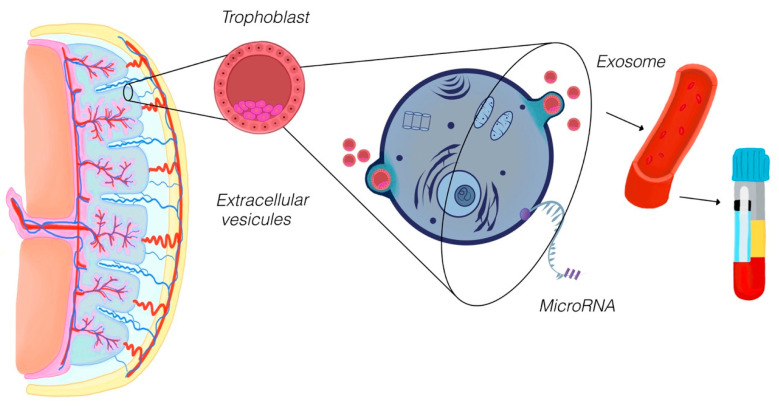
Extracellular vesicles (EVs) as molecular mediators in placental health.

EVs were another promising focus of the reviewed studies (Figure 2). EVs are lipid-bound vesicles secreted by cells, carrying molecular cargo such as proteins, lipids, and nucleic acids. One study demonstrated that EVs derived from endometrial organoids contained specific microRNAs associated with successful implantation and pregnancy maintenance. These findings indicate that EVs play an essential role in maternal–fetal communication and could serve as biomarkers for early pregnancy health [20]. Another investigation identified placental protein 13 (PP13) encapsulated in circulating EVs as a potential marker for the severity of preeclampsia. PP13 was found to be elevated in pregnancies with severe preeclampsia, underscoring its potential utility in predicting disease progression and guiding clinical management [21].

Recent studies have also explored the role of exosomal microRNAs as diagnostic tools in placenta accreta spectrum (PAS) disorders. For instance, Muñoz et al. identified a set of circulating exosomal miRNAs—including miR-1246 and miR-1910-5p—that were significantly upregulated in PAS patients, implicating the activation of the MAPK and Rap1 pathways involved in trophoblast invasion and placental adherence [22]. Similarly, Timofeeva et al. proposed a diagnostic panel of cell-free miRNAs with high predictive value for PAS, including miR-106b-5p and miR-519d-3p, further supporting the utility of liquid biopsy in the early, non-invasive detection of invasive placental disorders [23]. These findings expand the diagnostic potential of extracellular vesicle and cfRNA profiling beyond preeclampsia, underscoring their broader relevance in placental pathologies.

Despite their considerable promise, the application of liquid biopsy technologies in routine clinical settings faces several challenges. There is a need to improve the diagnostic precision of these tools through the development of standardized protocols for biomarker analysis. Furthermore, pre-analytical variables, such as sample handling and storage, must be optimized to ensure consistent and reliable results. Advances in bioinformatics and machine learning are anticipated to enhance the diagnostic accuracy and predictive capabilities of liquid biopsy, ultimately facilitating its integration into clinical practice for personalized perinatal care.

The figure illustrates the release and analysis of EVs, including exosomes, as pivotal components in maternal–fetal communication and perinatal research. During pregnancy, trophoblast cells within the placenta secrete EVs into the maternal bloodstream. These vesicles encapsulate diverse molecular cargo, such as microRNAs, proteins, and lipids, which reflect the physiological and pathological states of the placenta. The EVs are collected through maternal blood sampling and analyzed to uncover key molecular signals involved in processes like trophoblast invasion, immune modulation, and vascular remodeling. In this scoping review, EVs are highlighted as emerging biomarkers for early detection and monitoring of pregnancy complications, including preeclampsia, fetal growth restriction, and implantation disorders, offering significant potential for improving diagnostic precision and therapeutic strategies in perinatology.

### 3.4. Applications of Single-Cell Analysis in Perinatal Research

**Figure 3:**
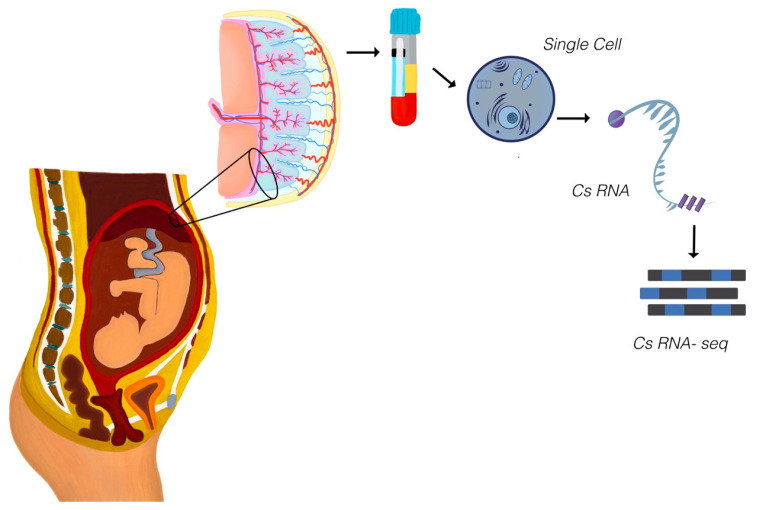
Single-cell RNA sequencing (scRNA-seq) for placental health analysis.

Single-cell analysis and organoid models have provided unprecedented insights into the cellular and molecular complexity of the placenta, offering a deeper understanding of maternal–fetal interactions and their implications for pregnancy complications (Figure 3). These technologies enable the characterization of individual cell populations, revealing critical pathways involved in normal and pathological conditions. This section highlights how single-cell technologies have revealed cell-type-specific mechanisms underpinning common obstetric complications, with emphasis on vascular remodeling, immune dysfunction, and cellular heterogeneity in preeclampsia and fetal growth restriction.

Trophoblast organoids, for example, have been developed to mimic early placental development and function. These organoids replicate key features of the placenta, such as hormone production, nutrient transport, and cellular invasion, providing a controlled environment for studying maternal–fetal interactions. One study used trophoblast organoids to investigate how placental cells differentiate and interact with maternal tissues, shedding light on mechanisms essential for maintaining a healthy pregnancy. This model has also proven valuable for exploring the pathophysiology of placental disorders, offering new avenues for therapeutic intervention [24]. scRNA-seq has emerged as a powerful tool for dissecting the cellular composition and molecular pathways of the placenta. One study employed scRNA-seq to analyze placental pericytes, identifying specific gene expression patterns associated with vascular remodeling. This process is critical for supporting fetal growth and ensuring adequate blood flow to the developing fetus. Seminal scRNA-seq studies of first-trimester placentas have identified specialized cell–cell interactions between extravillous trophoblasts, decidual stromal cells, and uterine NK cells, essential for proper spiral artery remodeling [25]. Disruptions in these pathways have been linked to conditions such as fetal growth restriction, highlighting the potential of scRNA-seq to uncover the molecular underpinnings of pregnancy complications [26]. scRNA-seq on placental tissues from early-onset preeclampsia pregnancies has unveiled molecular subtypes characterized by heightened myeloid inflammatory activity and endothelial cell dysfunction, distinguishing them from normotensive controls [27]. Another study utilized scRNA-seq to investigate neonatal endothelial cells, revealing disrupted signaling pathways in preterm infants with bronchopulmonary dysplasia. These findings underscore the utility of single-cell technologies in identifying cellular and molecular targets for intervention in both maternal and neonatal complications [28].

While the potential applications of single-cell analysis are vast, its integration into clinical practice remains limited by technical and economic barriers. The sensitivity of scRNA-seq must be improved to ensure accurate detection of rare cell populations, and protocols need to be streamlined to make the technology more accessible. Additionally, the high cost of single-cell analysis presents a significant obstacle, particularly in low-resource settings. Continued technological advancements are expected to address these limitations, paving the way for broader adoption of single-cell analysis in perinatal research and clinical care.

The figure demonstrates the process of single-cell RNA (scRNA) release and analysis as a cutting-edge approach in perinatal research. During pregnancy, the placenta releases RNA molecules from individual cells into the maternal bloodstream, reflecting the diverse cellular activities within placental tissues. These unicellular RNA fragments are collected via maternal peripheral blood sampling and processed using advanced sequencing technologies to generate high-resolution gene expression profiles. This technique enables the characterization of specific placental cell populations, providing insights into critical processes such as trophoblast differentiation, vascular remodeling, and immune interactions. In this scoping review, scRNA-seq is highlighted as an emerging tool for identifying cellular pathways and molecular mechanisms underlying pregnancy complications, such as preeclampsia, fetal growth restriction, and preterm birth, offering unprecedented opportunities for precision diagnostics and therapeutic interventions.

### 3.5. Limitations and Challenges

Liquid biopsy and single-cell analysis have opened new frontiers in perinatal research, yet several challenges limit their transition into clinical practice. High costs, limited access to equipment, and the absence of standardized protocols for sample handling and data analysis hinder their routine use. Additionally, the complexity of the data generated, particularly with single-cell technologies, demands advanced bioinformatics and specialized personnel, which are not always available in clinical settings.

While this review focuses on mapping current evidence rather than assessing clinical feasibility, it is important to highlight translational barriers. For instance, isolating EVs, including exosomes, often requires high-speed ultracentrifugation and technical expertise, limiting their practical application. Although alternative methods are emerging, issues of scalability and reproducibility persist. Acknowledging these limitations is essential to understanding the gap between promising research and clinical implementation.

Furthermore, the clinical translation of these technologies varies significantly depending on the specific application. For example, cell-free RNA analysis has already shown promise in early prediction of preeclampsia, with high accuracy in prospective cohorts, while single-cell RNA sequencing remains largely limited to mechanistic studies and lacks standardization for clinical diagnostics. Extracellular vesicle analysis faces technical and scalability challenges, and trophoblast organoids, though valuable for modeling placental diseases, are still far from routine application. Clear regulatory frameworks, cost-effectiveness analyses, and robust multicenter validations are urgently needed to bridge the translational gap. Without these efforts, even the most innovative tools may remain confined to research settings.

Moreover, both technologies face challenges related to sensitivity, reproducibility, and biological variability. In liquid biopsy, the low abundance and instability of cfRNA and EVs can compromise detection, particularly in early gestation or in conditions with subtle molecular signatures. Single-cell platforms, while powerful, are sensitive to sampling bias and often underrepresent rare or fragile placental cell populations. The absence of standardized data processing pipelines further complicates reproducibility across studies. Finally, ethical considerations surrounding omics-based testing in pregnancy—including incidental findings and data privacy—must be addressed through clear regulatory guidance before routine clinical integration becomes feasible.

## 4. Discussion

### 4.1. Interpretation of Findings

The findings of this review underscore the transformative role of emerging technologies in reshaping perinatal research and care. By offering unprecedented access to molecular and cellular details of the placenta, these tools address critical gaps in understanding pregnancy complications. Liquid biopsy, with its ability to detect dynamic molecular changes non-invasively, and single-cell analysis, which elucidates cellular complexity at unparalleled resolution, together form a foundation for advancing maternal and fetal health.

Beyond specific markers such as cfDNA and cfRNA, the broader implication of these technologies lies in their ability to bridge the gap between molecular discoveries and clinical applications. They enable researchers to move from observing phenomena to understanding mechanisms, paving the way for innovative diagnostic frameworks and personalized interventions. This paradigm shift signals a move toward precision perinatology, where care is tailored to the molecular profile of both the mother and fetus.

### 4.2. Strengths and Limitations

This review captures a broad spectrum of technological applications, synthesizing findings across studies to provide a comprehensive overview of their potential in perinatal care. The ability to integrate basic science with clinical relevance is a key strength, as it highlights the translational potential of these tools.

However, some limitations must be acknowledged. The exclusion of non-English studies may restrict the global applicability of the findings, particularly in regions with significant contributions to this field. Furthermore, the rapid pace of technological development means that some aspects of this review may quickly become outdated. Variability in methodological quality across the included studies also limits the generalizability of certain conclusions. Addressing these gaps will require continuous updates and a focus on standardizing methods across research settings.

### 4.3. Implications for Clinical Practice

The integration of these technologies into clinical practice has the potential to revolutionize perinatal care. Liquid biopsy offers a non-invasive, real-time approach to assessing placental function, allowing clinicians to monitor pregnancies dynamically and detect risks earlier than ever before. For example, cfRNA and cfDNA can provide critical insights into placental dysfunction, enabling timely interventions for high-risk pregnancies. However, the true promise of these technologies lies not only in diagnosis but in their ability to guide personalized care.

Despite these advancements, challenges remain. Implementing such advanced tools in routine practice requires significant investment in infrastructure, training, and resources, particularly in low-resource settings. Ensuring equity in access and affordability will be critical to realizing the full potential of these technologies.

### 4.4. Broader Implications for Research and Technology Development

The impact of these technologies extends beyond immediate clinical applications. They provide an unprecedented platform for addressing fundamental questions in placental biology and maternal–fetal health. For example, the ability to analyze EVs or resolve cellular interactions with single-cell technologies enables researchers to uncover mechanisms underlying trophoblast invasion, vascular remodeling, and immune modulation.

Emerging fields such as artificial intelligence (AI) offer additional opportunities to enhance these technologies. Integrating AI with molecular data from liquid biopsy and single-cell analysis can uncover complex patterns that are difficult to discern through traditional methods. These innovations have the potential to revolutionize risk prediction models, improve diagnostic accuracy, and facilitate personalized monitoring strategies. However, ensuring data integrity and addressing ethical concerns related to AI applications in healthcare will be critical moving forward.

The analysis of EVs, particularly exosomes, has emerged as a valuable tool for identifying biomarkers associated with implantation and the severity of preeclampsia, as evidenced by studies retrieved in our review. More recently, this approach has also shown promise in elucidating the molecular pathways involved in PAS. Alterations in exosomal miRNA profiles have been linked to the dysregulation of signaling cascades central to abnormal trophoblast adhesion and invasion, including MAPK, PI3K-Akt, and Rap1 pathways. These findings not only support the development of non-invasive diagnostic biomarkers but also provide a mechanistic window into the pathophysiology of PAS. Expanding the application of liquid biopsy to invasive placental disorders thus represents both a pressing research opportunity and a promising avenue for clinical innovation.

### 4.5. Recommendations for Future Research

To harness the full potential of these technologies, future research should focus on overcoming existing barriers. Standardizing protocols for cfDNA and cfRNA analysis is essential to ensure consistency and reliability across clinical and research settings. Similarly, single-cell analysis requires simplification and cost reduction to make it more accessible.

Expanding study populations to include diverse demographic and geographic groups will enhance the generalizability of findings and ensure equitable application. Longitudinal studies are needed to explore the long-term impact of these technologies on maternal and fetal outcomes, providing evidence for their integration into clinical guidelines. Finally, the exploration of synergies between liquid biopsy, single-cell technologies, and AI should remain a priority, as this intersection holds promise for creating transformative diagnostic and therapeutic tools.

### 4.6. Addressing Disparities and Equity

The promise of emerging technologies in perinatal care must be carefully weighed against the persistent disparities in access across different populations and regions. Socioeconomic and geographic factors continue to influence maternal and fetal health outcomes, and without deliberate efforts, novel tools may inadvertently widen existing inequities. To ensure inclusive progress, it is essential to promote the integration of these innovations into public health systems—particularly in underserved areas—through strategies such as cost subsidization, provider training, and the inclusion of diverse populations in research and clinical trials.

One of the most significant barriers to equitable implementation is the high cost and technical complexity of these technologies. As summarized in Table 2, per-sample costs vary widely: cfDNA testing, already in clinical use, ranges from approximately USD 350 to USD 795 [29], while experimental approaches like cfRNA analysis and EV profiling can cost between USD 500 and USD 1000 per sample. More advanced tools, such as scRNA-seq, often exceed USD 2000 per sample due to their specialized infrastructure and computational demands [30]. Similarly, organoid-based assays, although valuable for modeling placental development, remain costly and labor-intensive [31,32].

These financial and logistical challenges not only limit the adoption of these tools in low-resource settings but also underscore the need for scalable, affordable platforms tailored to diverse healthcare environments. Recognizing the economic dimension of technological innovation is critical to designing implementation pathways that are not only scientifically sound but also socially equitable. Promoting global collaboration and investment in capacity-building can help bridge these gaps, ensuring that the benefits of perinatal innovation reach all mothers and children, regardless of geography or socioeconomic status.

## 5. Conclusions

Liquid biopsy and single-cell analysis are transforming perinatal research by providing non-invasive methods to understand placental biology and address complications like preeclampsia and fetal growth restriction. These technologies bridge molecular discoveries with clinical applications, enabling earlier detection and personalized interventions. While challenges such as cost, accessibility, and standardization persist, continued innovation and equitable implementation will ensure their integration into clinical practice, improving outcomes for mothers and children worldwide.

## Figures and Tables

**Table 1:** Key applications and findings of emerging technologies in perinatal research.

Technology	Applications	Findings	Associated Pregnancy Complication(s)	Limitations
Liquid Biopsy	Non-invasive detection of preeclampsia, preterm birth, fetal growth restriction, and PAS through cfRNA, cfDNA, and EV profiling.	cfRNA: Inflammation-associated transcripts (e.g., *TNFSF4*) as early markers of preterm birth. cfDNA: Fragmentomics reflect placental dysfunction. EVs: microRNAs and proteins related to implantation and preeclampsia.	Preterm birth Preeclampsia FGR	Lack of standardized biomarker protocols. Pre-analytical variability (e.g., sample handling). High cost and limited accessibility.
EVs	Biomarker discovery for preeclampsia and PAS via circulating microRNAs and placental proteins.	PP13 in EVs: Marker of preeclampsia severity. Endometrial EV miRNAs linked to implantation. miR-1246, miR-1910-5p in PAS.	Preeclampsia PAS	Limited validation in clinical practice. Optimization needed for reliable biomarker isolation.
scRNA-seq	High-resolution analysis of cellular alterations in preeclampsia, FGR, and preterm birth using placental biopsies.	Disrupted pericyte and endothelial pathways in FGR and preeclampsia. Myeloid and endothelial signatures in early-onset preeclampsia.	Preeclampsia FGR Preterm birth	Requires fresh tissue and bioinformatics expertise; high cost.
Organoid Models	Functional modeling of trophoblast invasion and placental dysfunction in early-onset preeclampsia and implantation failure.	Trophoblast organoids replicate hormone production, nutrient transport, and invasive behavior.	Preeclampsia	Limited scalability for broader research. Cost and complexity in creating and maintaining organoids.

EVs—extracellular vesicles; scRNA-seq—single-cell analysis; FGR—fetal growth restriction; PAS—placenta accrete spectrum.

**Table 2:** Approximate costs and implementation barriers of emerging technologies in perinatal research.

Technology	Estimated Cost per Sample	Main Applications	Key Implementation Barriers
cfDNA	USD 350–795	Non-invasive prenatal testing; placental gene expression	Already commercialized; limited access in low-resource settings
cfRNA	USD 500–1000+	Inflammatory markers; early detection of preterm labor	Experimental; lacks standardization and clinical validation
EVs	USD 200–1000+	Biomarkers for implantation, preeclampsia	Requires ultracentrifugation or specialized platforms; low scalability
scRNA-seq	USD 1000–3000+	Placental cell-type profiling; pathway analysis	High costs; bioinformatics expertise and infrastructure needed
Organoid Models	Variable; ~USD 1000+ per assay	Functional modeling of placental development and pathology	Costly, labor-intensive; not scalable for clinical use

cfDNA—cell-free DNA; cfRNA—cell-free RNA; EVs—extracellular vesicles; scRNA-seq—single-cell RNA sequencing.

## Data Availability

The data employed for conducting this narrative review are available upon request to the following e-mail: torresmm@gmail.com.

## Supplementary Materials

The following supporting information can be downloaded at https://www.mdpi.com/article/10.3390/diagnostics15162056/s1, Figure S1. PRISMA flow diagram; Table S1: Search strategies; Table S2: Preferred Reporting Items for Systematic reviews and Meta-Analyses extension for Scoping Reviews (PRISMA-ScR) Checklist; Table S3: Summary of included studies.
