# Supplementary material for: Liquid Biopsy and Single-Cell Technologies in Maternal–Fetal Medicine: A Scoping Review of Non-Invasive Molecular Approaches
Source: Diagnostics (Basel). 2025 Aug 16;15(16):2056. doi: 10.3390/diagnostics15162056 (PMC12385971; doi:10.3390/diagnostics15162056)
Supplement: Supplementary file 1 [file diagnostics-15-02056-s001.zip › Figure S1. PRISMA Scoping.pdf]

Figure S1. PRISMA flow diagram

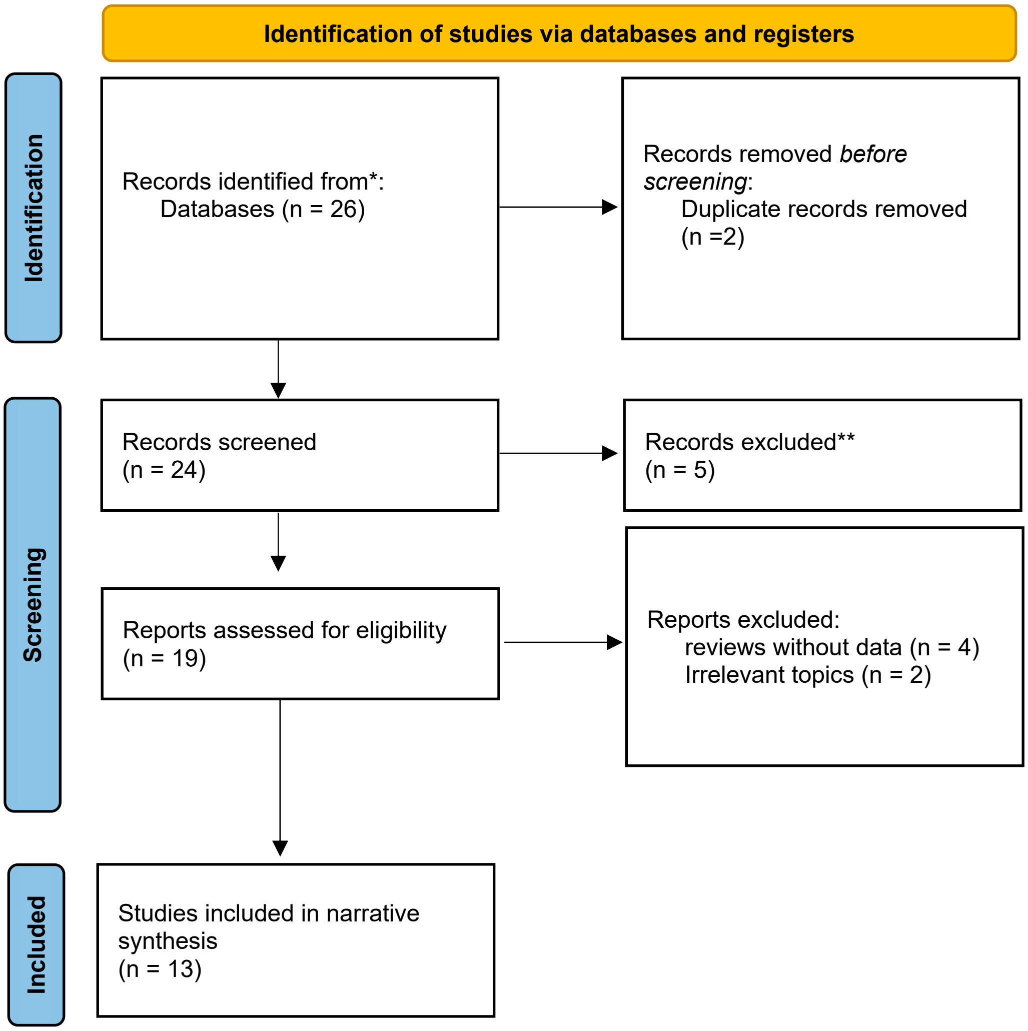

*Consider, if feasible to do so, reporting the number of records identified from each database or register searched (rather than the total number across all databases/registers).

**If automation tools were used, indicate how many records were excluded by a human and how many were excluded by automation tools.
