# Supplementary material for: Liquid Biopsy and Single-Cell Technologies in Maternal–Fetal Medicine: A Scoping Review of Non-Invasive Molecular Approaches
Source: Diagnostics (Basel). 2025 Aug 16;15(16):2056. doi: 10.3390/diagnostics15162056 (PMC12385971; doi:10.3390/diagnostics15162056)
Supplement: Supplementary file 1 [file diagnostics-15-02056-s001.zip › Table S1 Scoping.pdf]

**Table S1.** Search Strategies	
Database	Search Terms
PubMed	(("Perinatal Complications" OR "Pregnancy Complications" OR "Placental Disorders" OR "Preeclampsia" OR "Preterm Birth" OR "Preterm Labor" OR "Intrauterine Growth Restriction" OR "Fetal Growth Retardation" OR "Gestational Hypertension" OR "Placental Insufficiency" OR "Placental Development" OR "Maternal-Fetal Exchange" OR "Fetal Development" OR "Fetal Growth" OR "Perinatal Outcomes" OR "Obstetric Complications")<br>AND<br>("Liquid Biopsy" OR "Cell-Free DNA" OR "cfDNA" OR "cfRNA" OR "Circulating Tumor Cells" OR "Non-Invasive Prenatal Testing" OR "Prenatal Screening" OR "Cell-Free Fetal DNA" OR "Circulating Biomarkers" OR "Circulating DNA" OR "Circulating RNA" OR "Exosomes" OR "Extracellular Vesicles" OR "DNA Methylation" OR "Epigenetic Biomarkers")<br>AND<br>("Single Cell Analysis" OR "Single-Cell RNA Sequencing" OR "scRNA-seq" OR "Single-Cell Sequencing" OR "Single-Cell Proteomics" OR "Cellular Heterogeneity" OR "Organoids" OR "Placental Organoids" OR "3D Culture" OR "Organoids Culture" OR "Stem Cell-Derived Organoids" OR "Organ-on-a-Chip" OR "Placenta-on-a-Chip" OR "Advanced Diagnostic Techniques" OR "3D Model" OR "Placental Model" OR "Stem Cell Model" OR "Organoid Model"))
Scopus	(TITLE-ABS-KEY(("Perinatal Complications" OR "Pregnancy Complications" OR "Placental Disorders" OR "Preeclampsia" OR "Preterm Birth" OR "Preterm Labor" OR "Intrauterine Growth Restriction" OR "Fetal Growth Retardation" OR "Gestational Hypertension" OR "Placental Insufficiency" OR "Placental Development" OR "Maternal-Fetal Exchange" OR "Fetal Development" OR "Fetal Growth" OR "Perinatal Outcomes" OR "Obstetric Complications"))<br>AND<br>("Liquid Biopsy" OR "Cell-Free DNA" OR "cfDNA" OR "cfRNA" OR "Circulating Tumor Cells" OR "Non-Invasive Prenatal Testing" OR "Prenatal Screening" OR "Cell-Free Fetal DNA" OR "Circulating Biomarkers" OR "Circulating DNA" OR "Circulating RNA" OR "Exosomes" OR "Extracellular Vesicles" OR "DNA Methylation" OR "Epigenetic Biomarkers")<br>AND<br>("Single Cell Analysis" OR "Single-Cell RNA Sequencing" OR "scRNA-seq" OR "Single-Cell Sequencing" OR "Single-Cell Proteomics" OR "Cellular Heterogeneity" OR "Organoids" OR "Placental Organoids" OR "3D Culture" OR "Organoids Culture" OR "Stem Cell-Derived Organoids" OR "Organ-on-a-Chip" OR "Placenta-on-a-Chip" OR "Advanced Diagnostic Techniques" OR "3D Model" OR "Placental Model" OR "Stem Cell Model" OR "Organoid Model")))
Web of Science	TS=(("Perinatal Complications" OR "Pregnancy Complications" OR "Placental Disorders" OR "Preeclampsia" OR "Preterm Birth" OR "Preterm Labor" OR "Intrauterine Growth Restriction" OR "Fetal Growth Retardation" OR "Gestational Hypertension" OR "Placental Insufficiency" OR "Placental Development" OR "Maternal-Fetal Exchange" OR "Fetal Development" OR "Fetal Growth" OR "Perinatal Outcomes" OR "Obstetric Complications")<br>AND

	("Liquid Biopsy" OR "Cell-Free DNA" OR "cfDNA" OR "cfRNA" OR "Circulating Tumor Cells" OR "Non-Invasive Prenatal Testing" OR "Prenatal Screening" OR "Cell-Free Fetal DNA" OR "Circulating Biomarkers" OR "Circulating DNA" OR "Circulating RNA" OR "Exosomes" OR "Extracellular Vesicles" OR "DNA Methylation" OR "Epigenetic Biomarkers")<br>AND<br>("Single Cell Analysis" OR "Single-Cell RNA Sequencing" OR "scRNA-seq" OR "Single-Cell Sequencing" OR "Single-Cell Proteomics" OR "Cellular Heterogeneity" OR "Organoids" OR "Placental Organoids" OR "3D Culture" OR "Organoids Culture" OR "Stem Cell-Derived Organoids" OR "Organ-on-a-Chip" OR "Placenta-on-a-Chip" OR "Advanced Diagnostic Techniques" OR "3D Model" OR "Placental Model" OR "Stem Cell Model" OR "Organoid Model")))
