# Supplementary material for: Liquid Biopsy and Single-Cell Technologies in Maternal–Fetal Medicine: A Scoping Review of Non-Invasive Molecular Approaches
Source: Diagnostics (Basel). 2025 Aug 16;15(16):2056. doi: 10.3390/diagnostics15162056 (PMC12385971; doi:10.3390/diagnostics15162056)
Supplement: Supplementary file 1 [file diagnostics-15-02056-s001.zip › Table S3.pdf]

**Table S3.** Summary of included studies					
**No.**	**Title**	**Authors**	**Year**	**Journal**	**DOI**
1	DNA methylation landscape in pregnancy-induced hypertension: progress and challenges.	Deng F, Lei J, Qiu J, Zhao C, Wang X, Li M Zhang, Q Gao.	2024	Reproductive Biology and Endocrinology	DOI: 10.1186/S12958-024-01248-0
2	Basic Science with Preclinical Models to Investigate and Develop Liquid Biopsy: What Are the Available Data and Is It a Fruitful Approach?	Cena B, Melloul E, Demartines N, Dormond O, Labgaa I.	2022	International Journal of Molecular Sciences	DOI: 10.3390/IJMS23105343
3	Increased nuchal translucency after low-risk noninvasive prenatal testing: What should we tell prospective parents?	Kelley J, McGillivray G, Meagher S, Hui L.	2021	Prenatal Diagnosis	DOI: 10.1002/PD.6024
4	Genome-wide miRNA profiling in plasma of pregnant women with down syndrome fetuses.	Zedníková I, Chylíková B, Šeda O, Korabečná M, Pazourková E, Břešťák M, et al.	2020	Molecular Biology Reports	DOI: 10.1007/S11033-020-05545-W
5	Liquid Biopsy Based on Cell-Free DNA and RNA.	Loy C, Ahmann L, De Vlaminck I, Gu W.	2024	Annu Rev Biomed Eng	DOI: 10.1146/annurev-bioeng-110222-111259
6	Early prediction of preeclampsia in pregnancy with cell-free RNA.	Moufarrej MN, Vorperian SK, Wong RJ, Campos AA, Quaintance CC, Sit RV, et al.	2022	Natura	DOI:10.1038/s41586-022-04410-z
7	Investigating Pregnancy and Its Complications Using Circulating Cell-Free RNA in Women's Blood During Gestation.	Moufarrej MN, Wong RJ, Shaw GM, Stevenson DK, Quake SR.	2022	Front Pediatr.	DOI:10.3389/fped.2020.605219

8	Circulating extracellular vesicles in healthy and pathological pregnancies: A scoping review of methodology, rigour and results.	Barnes MVC, Pantazi P, Holder B.	2023	Extracell Vesicles	DOI: 10.1002/jev2.12377
9	Cell-Free Nucleic Acids for Early Prediction of Preeclampsia.	Moufarrej MN, Winn VD, Quake SR.	2024	Curr Hypertens Rep	DOI: 10.1007/s11906-023-01291-z
10	Single-cell reconstruction of the early maternal-fetal interface in humans.	Vento-Tormo R, Efremova M, Botting RA, Turco MY, Vento-Tormo M, Meyer KB, et al.	2018	Nature	DOI: 10.1038/s41586-018-0698-6
11	Single-cell transcriptional profiling reveals cellular and molecular divergence in human maternal-fetal interface.	Wang Q, Li J, Wang S, Deng Q, An Y, Xing Y, et al.	2022	Sci Rep	DOI: 10.1038/s41598-022-14516-z
12	Human placental biology at single-cell resolution: a contemporaneous review.	Barrozo ER, Aagaard KM.	2022	Bjog.	DOI: 10.1111/1471-0528.16970
13	Single cell transcriptome research in human placenta.	Li H, Huang Q, Liu Y, Garmire LX.	2020	Reproduction	DOI: 10.1530/REP-20-0231
14	Single cell RNA sequencing research in maternal fetal interface.	Chen Q, Shan D, Xie Y, Luo X, Wu Y, Chen Q, et al.	2022	Front Cell Dev Biol.	DOI: 10.3389/fcell.2022.1079961
15	review of the application of single-cell RNA sequencing in pregnancy-related diseases.	Zhou Z, Yang X. An update	2024	Front Endocrinol	DOI: 10.3389/fendo.2024.1415173
16	Revealing the molecular landscape of human placenta: a systematic review and meta-analysis of single-cell RNA sequencing studies.	Derisoud E, Jiang H, Zhao A, Chavatte-Palmer P, Deng Q.	2024	Hum Reprod	DOI: 10.1093/humupd/dmae006

17	The increased cfRNA of TNFSF4 in peripheral blood at late gestation and preterm labor: its implication as a noninvasive biomarker for premature delivery.	Wang Z, Ou Q, Gao L.	2023	Frontiers in Inmunology	DOI:10.3389/FIMMU.2023.1154025
18	Non-invasive determination of gene expression in placental tissue using maternal plasma cell-free DNA fragmentation characters.	Li K, Guo Z, Li F, Lu S, Zhang M, Gong Y, et al.	2024	Gene	DOI: 10.1016/j.gene.2024.148789
19	A differentiation roadmap of murine placentation at single-cell resolution.	Jiang X, Wang Y, Xiao Z, Yan L, Guo S, Wang Y, et al.		Cell Discov	DOI: 10.1038/s41421-022-00513-z
20	Extracellular vesicles secreted by adenomyosis endometrial organoids contain miRNAs involved in embryo implantation and pregnancy.	Juárez-Barber E, Segura-Benítez M, Carbajo-García MC, Bas-Rivas A, Faus A, Vidal C, et al.	2023	Reprod Biomed Online	DOI: 10.1016/J.RBMO.2022.12.008
21	Augmented Placental Protein 13 in Placental-Associated Extracellular Vesicles in Term and Preterm Preeclampsia Is Further Elevated by Corticosteroids.	Kazatsker MM, Sharabi-Nov A, Meiri H, Sammour R, Sammar M	2023	International Journal of Molecular Sciences	DOI: 10.3390/IJMS241512051
22	Serum Exosomal MicroRNA Pathway Activation in Placenta Accreta Spectrum: pathophysiology and detection.	Jessian L. Munoz, et al.	2024	AJOG	DOI: 10.1016/j.xagr.2024.100319
23	Diagnostic Role of Cell-Free miRNAs in Identifying Placenta Accreta Spectrum.	Angelika V. Timofeeva, et al.	2024	International Journal of Molecular Sciences	DOI: 10.3390/ijms25020871

24	Trophoblast organoids as a model for maternal-fetal interactions during human placentation.	Turco MY, Gardner L, Kay RG, Hamilton RS, Prater M, Hollinshead MS, et al	2018	Nature	DOI: 10.1038/S41586-018-0753-3
25	Single-cell RNA-seq reveals cell type-specific transcriptional signatures at the maternal-foetal interface during pregnancy.	Nelson AC, Mould AW, Bikoff EK, Robertson EJ.		Nat Commun	DOI: 10.1038/ncomms11414
26	Gene expression profile of human placental villous pericytes in the first trimester – An analysis by single-cell RNA sequencing.	Liu Z	2024	Reproductive Biology	DOI: 10.1016/J.REPBIO.2024.100919
27	Single-nuclei RNA-sequencing fails to detect molecular dysregulation in the preeclamptic placenta.	Admati I, Skarbianskis N, Hochgerner H, Ophir O, Yagel S, Solt I, et al.	2025	Placenta	DOI: 10.1016/j.placenta.2024.12.011
28	The vascular phenotype of BPD: new basic science insights—new precision medicine approaches.	Durlak W, Thébaud B.	2024	Pediatric Research	DOI: 10.1038/S41390-022-02428-7
29	Clinical utility and cost of non-invasive prenatal testing with cfDNA analysis in high-risk women based on a US population.	Song K, Musci TJ, Caughey AB.	2013	J Matern Fetal Neonatal Med	DOI: 10.3109/14767058.2013.770464
30	A Cost-Effectiveness Analysis of First Trimester Non-Invasive Prenatal Screening for Fetal Trisomies in the United States.	Walker BS, Nelson RE, Jackson BR, Grenache DG, Ashwood ER, Schmidt RL.	2015	PLoS One	DOI: 10.1371/journal.pone.0131402
31	Comparison of the Cost and Effect of	Chang YH, Wu KC,	2023	Cell Transplant	DOI: 10.1177/09636897231160216

	Combined Conditioned Medium and Conventional Medium for Fallopian Tube Organoid Cultures.	Harnod T, Ding DC			
32	Methodologies for Generating Brain Organoids to Model Viral Pathogenesis in the CNS.	Hopkins HK, Traverse EM, Barr KL.	2021	Pathogens	DOI: 10.3390/pathogens10111510
